# Effect of cooking and preservation on nutritional and phytochemical composition of the mushroom *Amanita zambiana*

**DOI:** 10.1002/fsn3.428

**Published:** 2016-09-28

**Authors:** Tsungai Reid, Merjury Munyanyi, Takafira Mduluza

**Affiliations:** ^1^ Biochemistry DepartmentUniversity of ZimbabweHarareZimbabwe; ^2^ School of Laboratory Medicine & Medical SciencesUKZNDurbanSouth Africa

**Keywords:** *Amanita zambiana*, cooking, mushroom, nutrition, preservation

## Abstract

The effect of different cooking and preservation methods on the nutritional and phytochemical composition of the mushroom, *Amanita zambiana*, was investigated. Fresh mushrooms were boiled in water, fried, or microwaved. In addition, fresh mushrooms were either air‐dried for 7 days or frozen at −20°C for 14 days. The protein, lipid, carbohydrate, and phenolic content of the treated mushrooms were measured and compared to the fresh mushroom contents. Frying increased the protein (2.01% ± 0.2% [fresh mushroom] to 2.23% ± 0.09%), lipid (14.68% ± 0.9% to 15.56% ± 0.34%), and carbohydrate (0.89% ± 0.01% to 2.69% ± 0.03%) content, while microwaving increased the protein (2.01% ± 0.2% to 3.64% ± 0.08%) and carbohydrate content (0.89% ± 0.01% to 2.26% ± 0.09%). Boiling only increased the carbohydrate content (0.89% ± 0.01% to 1.71% ± 0.05%) of the mushroom and significantly decreased (*p* < .05) the phenolic content (8.77 ± 0.1 to 1.46 ± 0.2 mg gallic acid equivalent (GAE)/g mushroom). Drying resulted in significant increase (*p* < .05) in protein (2.01 ± 0.2% to 24.36 ± 0.09%), carbohydrate (0.89% ± 0.01% to 58.67% ± 3.29%), and phenolic contents (8.77 ± 0.1 to 119.8 ± 0.7 mg GAE/g mushroom), while freezing only increased the carbohydrate content (0.89% ± 0.01% to 1.77% ± 0.03%). From the three cooking methods studied, frying is recommended as the most effective cooking procedure in retaining or enhancing the mushroom nutrients, while drying is a better preservation method than freezing.

## 1 Introduction

Wild edible mushrooms have been a part of human diet in many regions of the world for centuries due to their pleasant taste and aroma as well as their nutritional values. Mushrooms contain large amounts of dietary fiber, proteins, vitamins, and minerals. A number of mushrooms are even consumed for medicinal purpose as they contain valuable bioactive compounds that include phenolic compounds. Phenolic compounds are secondary metabolites and are a potent source of antioxidants. Mushrooms are becoming an increasingly important source of food and medicinal purposes due to their phenolic and antioxidant contents (Wang & Xu, 2014; Caglarirmak, 2011; Kuka, Cakste, Galoburda, & Sabovics, 2014; Moon & Lo, 2013; Oktay et al., 2015).

Studies have shown that mushrooms contain proteins especially amino acids that are essential to human health. Mushrooms are rich in leucine and lysine amino acids, which are commonly lacking in many staple cereal foods. Proteins are essential for general growth, body tissue repair, and even maintenance of healthy cells. Some mushroom proteins have antibacterial and anticancer properties (Cheung, 2008; Wani, Bodha, & Wani, 2010; Xu et al., 2014). Crude protein content has been reported to vary from 10% to 40% in some of the common edible mushrooms (Barros, Cruz, Baptista, Estevinho, & Ferreira, 2008; Diez & Alvarez, 2001; Longvah & Deosthale, 1998). Carbohydrates are also an important component of mushrooms constituting between 50% and 60% of the dry matter. Mushroom carbohydrates have been found in many cases to have anticancer properties (Kalac, 2012). While fats increase palatability of foods by retaining and absorbing flavors, they are essential in diet (Shadung, Mphosi, & Mashela, 2012), even though low values of fats ranging from 2% to 6% of dry matter are required. Furthermore, fats are important in the biological and structural functioning of cells and help in transportation of fat‐soluble vitamins, which are nutritionally essential. Mushrooms have fat in their cell wall, which is important for storage of vitamin D. Mushrooms contain mainly the healthy unsaturated fats like oleic and linoleic acids, that are found at low levels but have beneficial value when consumed (Cheung, 2008).

*Amanita zambiana* is an edible mushroom which falls in the *Amanita* genus. The mushroom consists of a cap that is brown over the disk, paler toward margin, and white at the margin. *A. zambiana* is known as the Zambian Slender Caesar in English and like all *Amanita* species is mycorrhizal, associating with trees in the genus *Brachystegia*. The geographic distribution of *A. zambiana* is confined to South Central Africa, including southern Zaire (De Roman, 2010; Ryvarden, Piearce, & Masuka, 1994). Prior to its naming in 1980, the mushroom may have been referred to as the closely related, south Asian species *A. hemibapha*. A survey of *Amanita* species in sub‐Saharan Africa indicates that the species described as *Amanita loosii* from Zaire represent an earlier name for the species (Harkonen, 2002; Sharp, 2011). The mushroom is an early season species locally dubbed the Christmas mushroom, although it may also appear in a brief secondary flush toward the end of the rains (Pegler & Piearce, 1980; Ryvarden et al., 1994). *A. zambiana* is one of the best known edible fungi throughout the region with masses of the mushrooms dominating roadside market stalls around December. The species, significantly contributes to the Zimbabwean household food security, when in season (Garwe, Munzara‐Chawira, & Kusena, 2009; Ryvarden et al., 1994).

In Zimbabwe, mushrooms are commonly cooked before being consumed or they are preserved either by drying or freezing before cooking. Different preservation and cooking methods have been found to have a significant effect on the final nutritional composition of different mushrooms (Aishah & Rosli, 2013; Kumar, Singh, & Singh, 2013; Pogon, Jaworsaka, Duda‐Chodak, & Maciejaszek, 2013). Preservation is essential for extension of shelf life and for value addition. Studies have shown that preservation may improve nutritional and sensory quality substantially, hence add value to mushrooms (Chelela, Chacha, & Matemu, 2014). There is lack of data available on how the different preservation and cooking methods affect the composition of locally edible mushrooms, including *A. zambiana*. This study therefore aimed at evaluating the effects of different cooking and preservation methods on the total proteins, lipids, carbohydrates, and phytochemicals of *A. zambiana*. The information contributes toward determining the best cooking and preservation method that retains most of the nutrients and phytochemicals in *A. zambiana*.

## 2 Materials and Methods

### 2.1 Sample collection and identification of the mushrooms

Fresh mushroom (*Amanita zambiana*) fruit bodies were collected from Marondera in Zimbabwe. Identification of the mushroom was done by comparing the photographical, morphological, and anatomical characteristics with the description given in the manuals on identification of mushrooms in Southern Africa (Ryvarden et al., 1994; Sharp, 2011).

### 2.2 Sample preparation

Fresh raw mushrooms were cleaned by running tap water, followed by distilled water, and then exposed to different cooking and preservation treatments.

#### 2.2.1 Boiling

Thirty grams of fresh mushroom pieces were placed in a pot with 300 ml of boiling water (ordinary cooking). Boiling was done until the mushroom was tender and the boiled mushroom was ground using a mortar and pestle.

#### 2.2.2 Microwaving

Thirty grams of mushroom pieces were placed in a glass dish and cooked in a microwave oven for 2 min at 900 watts. The microwaved mushroom sample was ground with a mortar and pestle.

#### 2.2.3 Frying

Thirty grams of mushroom pieces were placed in a frying pan with 100 ml of hot cooking oil while stirring until they became crisp tender. Cooking oil was removed by filtration and the sample was ground using a mortar and pestle.

#### 2.2.4 Air drying

Three hundred grams of fresh mushrooms were cut into small pieces, placed on a clean bond paper, and left to air dry for 7 days. The air‐dried mushroom sample was ground until the sample passed through a 0.1‐mm sieve.

#### 2.2.5 Freezing

Fifty grams of fresh mushroom fruit bodies were cut into small pieces, placed in sealable plastic bags, and frozen in a −20°C freezer for 14 days. The frozen samples were removed from the freezer, allowed to thaw, and ground using a mortar and pestle.

### 2.3 Nutritional analysis

#### 2.3.1 Determination of crude protein (Kjeldahl method)

One and a half grams of the air‐dried or 3.0 g of treated mushroom samples were digested in a Kjeldahl digestion flask by boiling with 25 ml of concentrated sulfuric acid and 10 g of Kjeldahl catalyst tablet until the mixture was clear. After cooling the flasks, 400 ml of cold water and 100 ml of 40% sodium hydroxide were added. The contents were distilled until 200 ml of solution was collected, and mixed with 50 ml of boric acid with indicator. The solution was titrated with 0.097 mol/L HCl which had been standardized with sodium carbonate. The protein content was calculated as follows:

$$\begin{array}{cc} \%\mathrm{Nitrogen}\;= & \;A\;\times \;\mathrm{acid}\;\mathrm{molarity}\;\times \;0.014\\ & \times \;100/\mathrm{weight}\;\mathrm{of}\;\mathrm{sample}\;\mathrm{in}\;\mathrm{grams}, \end{array}$$

where: A is the volume of 0.097 mol/L hydrochloric acid titrated minus volume of blank.

%Crude Protein (CP)=%Nitrogen×Protein Factor (4.38)

#### 2.3.2 Determination of crude lipid

Lipids were extracted from 5 g of mushroom samples for 4 hr with hexane using the Soxhlet apparatus, after which hexane was evaporated to dryness. The weight of crude lipid was obtained from the difference between the initial and the final weight.

#### 2.3.3 Determination of total carbohydrate

The total carbohydrate content was determined by the calorimetric method as described by Albalasmeh, Berhe, and Ghezzehei (2013) with modifications. Briefly, 2 g of ground mushroom was dissolved in 50 ml of water and centrifuged at 1000 rpm for 2 min. The mushroom extract was rapidly mixed in a test tube with 3 ml of concentrated sulfuric acid and vortexed for 30 s. After cooling, absorbance of the solution was read at 315 nm with a UV spectrophotometer. Glucose (ranging from 0 to 1 mg/ml) was used to construct a calibration curve (*R* ^2^ = 0.9982).

### 2.4 Phytochemical analysis

#### 2.4.1 Preparation of methanol extracts

Ground mushroom of 10 g was mixed with 40 ml of 98% methanol and the sample was placed in an incubator shaker at 180 rpm and 37°C for 48 hr. This extraction procedure was repeated for the fresh, frozen, and differently cooked mushroom samples. The frozen sample was allowed to thaw before being ground with a mortar and pestle. For the dried mushroom sample, 1 g was dissolved in 10 ml of 98% methanol and placed in an incubator shaker at 180 rpm and 37°C for 24 hr. All the extraction mixtures were filtered through Whatman No. 2 filter paper and the filtrate was used directly for analysis.

#### 2.4.2 Determination of total phenol

The total phenolic content was determined by the Folin–Ciocalteu calorimetric method as described by Barros, Baptista, Correia, Morais, and Ferreira (2007). A quantity of 1 ml of the filtrate was mixed with 1 ml of 10% v/v of Folin–Ciocalteu reagent, followed by 1 ml of 7.5% sodium carbonate. The mixture was adjusted to 10 ml with distilled water. The mixture was allowed to stand for 90 min in the dark and the absorbance was read at 725 nm. Gallic acid (ranging from 0 to 0.3 mg/ml) was used to construct the standard curve (*R* ^2^ = 0.9982) and the results were expressed as mg of gallic acid equivalents.

### 2.5 Statistical analysis

All the analyses were done in triplicate. The data were expressed graphically and as means ± standard deviation. Graph Pad Prism 5.03 software was used for statistical analysis of data and ANOVA (one‐way analysis of variance) was done. Bonferroni's multiple comparison tests were used for comparisons between means and the significant difference between means was accepted at *p* ≤ .05.

## 3 Results and Discussion

### 3.1 Effects of different preservation and cooking methods on crude protein content

**Figure 1:**
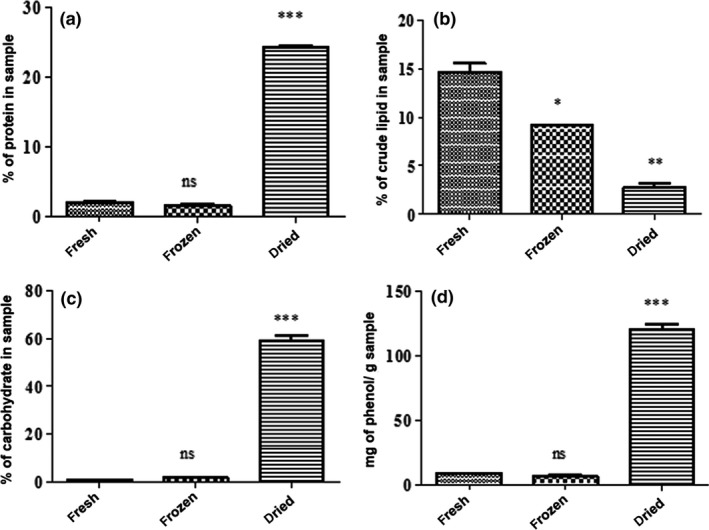
Effect of different preservation methods on: (a) crude protein content, (b) crude lipid content, (c) total carbohydrate content, and (d) total phenolic content of *A. zambiana* mushroom. ***p < .001, **p < .01 and *p <. 0001 compared to the fresh sample (one‐way ANOVA followed by Bonferroni's multiple comparison test). Results are expressed as ± standard deviation of three measurements

Mushrooms are seasonal, found mostly in the rainy season, and are highly perishable. Preservation is essential for extension of shelf life and for value addition. The dried, fresh, and frozen mushroom samples gave different protein content of 24.36% ± 0.09%, 2 .01% ± 0.19%, and 1.62% ± 0.16%, respectively. There was a significant increase in protein content (*p* < .05) with drying and no significant difference (*p* < .05) with freezing (Fig. 1a). An increase in proteins with drying might be due to the fact that drying removes water, hence concentrating the nutrients that are left. The increase in protein content obtained after drying is similar to the results obtained in other studies (Breene, 1990; Alam et al., 2007). Protein content of dried mushrooms ranges from 19% to 39% (Breene, 1990), similar to the value obtained in this study.

**Figure 2:**
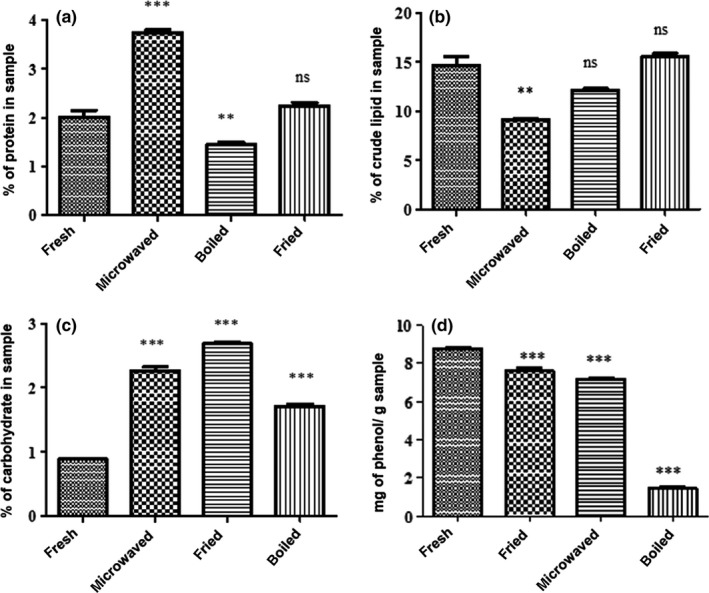
Effect of different cooking methods on: (a) crude protein content, (b) crude lipid content, (c) total carbohydrate content, and (d) total phenolic content of *A. zambiana* mushroom. ***p < .001 and **p < .01 compared to the fresh sample (one‐way ANOVA followed by Bonferroni's multiple comparison test). Results are expressed as ± standard deviation of three measurements

Consumption of wild edible mushrooms is increasing due to a good content of carbohydrates, fats, vitamins, minerals, and proteins, which contain all essential amino acids. Mushrooms have a good nutritional value, particularly as a source of protein that can enrich human diets, especially in developing countries where animal protein may not be available or costly (Colak, Faiz, & Sesli, 2009; Ezeibekwe, Ogbonnaya, Unamba, & Osuala, 2009). The boiled, fresh, microwaved, and fried mushroom samples gave different protein content of 1.62% ± 0.08%, 2.01% ± 0.2%, 3.64% ± 0.08%, and 2.23% ± 0.09%, respectively. There was a significantly high protein content (*p* < .05) in the microwaved sample compared to the fresh mushroom but there was a decrease in the boiled sample (Fig. 2a). The decrease in the boiled mushroom sample might be due to solubilization and leaching out of the nitrogenous substances during the boiling treatment. The increase in crude protein with microwaving and frying might be due to increase in protein availability as a result of enzyme hydrolysis of insoluble protein (Echendu, Obizoba, & Anyika, 2009). A similar trend was observed in studies done by Bliss (1975) who reported that the increase in protein could be a result of enzymatic hydrolysis which may cause release of free amino acids.

### 3.2 Effect of different preservation and cooking methods on crude lipid content

The fresh, frozen, and dried mushroom samples gave lipid content of 14.68% ± 0.9%, 9.16% ± 0.06%, and 2.8% ± 0.4%, respectively. There was a significant decrease (*p* < .05) in crude lipid content with all the preservation methods (Fig. 1b). According to Barros et al. (2007), dried samples of different mushroom species have lower total lipid composition than their corresponding fresh and frozen samples, similar to the trend found in this study. The decrease in the lipid content might be due to an increase in lipase activity and denaturation of the lipid fraction during the preservation treatment.

The results shown in Fig. 2b indicate the effect of different cooking methods on the lipid content of *A. zambiana*. The fresh, microwaved, fried, and boiled mushroom samples gave percentage lipid content of 14.68% ± 0.9%, 9.15% ± 0.13%, 15.56% ± 0.34%, and 12.12% ± 0.2%, respectively. There was a significant decrease (*p* < .05) in the lipid content in the microwaved mushroom sample. Kylen and McCready (1975) reported a similar trend of lipid decrease with cooked mushrooms. The decrease in lipids might be due to the denaturation and breakdown of the lipids into glycerol and fatty acids (Igbedioh, Olugbemi, & Akpapunan, 1994). There was no significant change (*p* < .05) in the boiled and fried samples compared to the fresh sample. The boiling method retained more of the lipids than microwaving which might be due to the extremely high temperatures in the microwaving treatment than the boiling temperature. Hence, more lipids were denatured during the microwaving treatment as compared to boiling.

### 3.3 Effect of different preservation and cooking methods on total carbohydrate content

The fresh, dried, and frozen mushroom samples gave carbohydrate content of 0.89% ± 0.01%, 58.67% ± 3.29%, and 1.77% ± 0.03%, respectively. There was a significant increase (*p* < .05) in total carbohydrate content with drying (Fig. 1c). The increase in carbohydrates with drying might be due to a decrease in moisture content, hence concentrating the nutrients. There was no significant increase (*p* < .05) with freezing. A similar trend was observed by Longvah and Deosthale (1998). The composition of edible mushroom carbohydrates ranges from 35% to 70% dry weight and varies with species (Diez & Alvarez, 2001) and the carbohydrate content of dried mushroom sample found in this study falls within the range.

The fresh, boiled, fried, and microwaved mushroom samples gave carbohydrate content of 0.89% ± 0.01%, 1.71% ± 0.05%, 2.69% ± 0.03%, and 2.26% ± 0.09%, respectively (Fig. 1c). There was a significant increase (*p* < .05) in carbohydrate content with all the cooking methods. The fried sample had a higher yield in carbohydrates followed by microwaving and boiling. The increase in carbohydrate content with cooking might be due to the destruction of mushroom cell walls that causes an increase in solubility of carbohydrates in water. A smaller increase of carbohydrates in boiled sample might be due to the fact that when the cell walls are destroyed, the carbohydrates might have leached into the boiling water before the extraction for analysis. The fried sample showed the highest increase, maybe because carbohydrates are insoluble in oil; hence, no leaching of carbohydrates during the cooking process.

### 3.4 Effect of different preservation and cooking methods on total phenolic content

The fresh, dried and frozen mushroom samples gave total phenolic content of 8.77 ± 0.1, 119.8 ± 0.7, and 6.58 ± 0.6 mg GAE/g mushroom, respectively (Fig. 1d). There was a significant increase (*p* < .05) in total phenolics with drying. This might be due to the changes in extractability of polyphenols as a result of plant cell wall destruction after drying treatment, thus bound polyphenols may be released more easily in the dried sample than the corresponding fresh sample. Drying may supply mushrooms with good enough energy to improve extraction without destroying the phenolic structures. There was no significant change (*p* < .05) in total phenolic content with freezing.

Phenolic compounds are bioactive compounds with lignin and flavonoid contents and are crucial due to the free radical scavenging activity. Bioactive compounds protect the body from reactive oxygen species that can cause cell damage (Kosanic, Rankovic, & Dasic, 2013; Oksana, Marian, Mahendra, & Bo, 2012). There exists consensus of various researchers that phenolics are the main antioxidants in mushrooms (Kalac, 2012). The total phenolics obtained for the fresh, microwaved, fried, and boiled mushroom samples were 8.77 ± 0.1, 7.17 ± 0.06, 7.62 ± 0.2, and 1.46 ± 0.2 mg GAE/g, respectively (Fig. 2d). There was a general decrease in total phenolic content with cooking. The reduction in phenolic content after cooking treatments might be due to the fact that some polyphenols are heat labile and are degraded upon heating (Rakic et al., 2007). The decrease was significantly higher (*p* < .05) in the boiled sample than all other cooking treatments done in this study. Greatest losses observed in the boiling treatment could be due to leaching out and denaturation of polyphenols. The decrease in total phenolics with cooking is similar to reports from other studies (Barros et al., 2007; Sun, Bai, & Zhuang,^2012^).

## 4 Conclusion

Proper preservation of food is important, making it available all season, but the preservation methods need to be appropriate in keeping the nutritional value of the food. Drying was shown to increase phenolics, proteins, carbohydrates, and decrease lipids, while freezing decrease lipids and causes little change in carbohydrates, phenolics, and proteins. Preservation was observed to have a positive impact on the nutrient and phytochemical composition of the mushrooms. Of the two preservation methods in this study, drying was found to significantly increase the amount of phenolics, carbohydrates, and proteins compared to freezing, hence is a better preservation method for mushrooms than freezing. The choice of food processing methods is also important in maintaining the nutritional values. In this study, microwaving was observed to increase the protein and carbohydrate content, while decreasing phenolics and lipids. Frying increased carbohydrates, proteins, and lipids, and decreased phenolics, while boiling decreased proteins, lipids, and phenolics, and increased carbohydrates. The boiling treatment retained lipids and lost proteins and phenolics more than any other cooking treatment and showed a relatively lower increase in carbohydrates. This means boiling results in the loss of most nutritional and phytochemical components, but retains lipids which are not needed by the body in large amounts, hence boiling is less recommended for mushroom *A. zambiana*. Microwaving increased proteins more than other cooking treatments, retained phenolics, and lost more lipids than the boiling treatment. However, frying increased more carbohydrates and lipids and retained more phenolics than other cooking treatments, while retaining more proteins than the boiling method. This makes frying a better way of cooking mushrooms as it retained most of the nutrients. This study therefore shows that, among the tested cooking methods, the best method of cooking mushrooms that retains or enhances nutritional benefits is frying followed by microwaving.

## Conflict of Interest

None declared.
